# *Euonymus maotaiensis* M.T.An & Xu Wu, sp. nov., a new species of *Euonymus* (Celastraceae) from southwest China

**DOI:** 10.3897/phytokeys.270.171340

**Published:** 2026-02-02

**Authors:** Xu Wu, Feng Liu, Ming-Tai An, Jiang-Hong Yu, Wan-Ying Yang

**Affiliations:** 1 College of Forestry, Guizhou University, Guiyang 550025, Guizhou, China College of Forestry, Guizhou University Guiyang China; 2 Key Laboratory of Plant Resource Conservation and Germplasm Innovation in Mountainous Region (Ministry of Education), College of Life Sciences/Institute of Agro-bioengineering, Guizhou University, Guiyang 550025, Guizhou, China College of Life Sciences/Institute of Agro-bioengineering, Guizhou University Guiyang China

**Keywords:** Celastraceae, *
Euonymus
*, Guizhou Province, new species, phylogenomic

## Abstract

This paper describes a new species of the genus *Euonymus* from Guizhou Province in southwestern China, *Euonymus maotaiensis*. The new species is morphologically similar to *E. chloranthoides* but can be distinguished by several key characteristics: broadly lanceolate leaves (as opposed to elliptic to oblong-elliptic), longer petioles (5–12 mm vs. 1–2 mm), longer peduncles (4–9 cm vs. 1–2 cm), 4- or 5-merous flowers (vs. strictly 5-merous), suborbicular petals with the upper half completely recurved abaxially (vs. planar petals), and indehiscent, 4–5 shallowly sulcate capsules (vs. 5-lobed capsules dehiscent to near the midpoint). These morphological differences are further supported by molecular phylogenetic analyses.

## Introduction

The genus *Euonymus* Linnaeus (Linnaeus 1753a) belongs to Celastraceae and comprises approximately 130 species that are primarily distributed in the Northern Hemisphere, with centers of diversity in East Asia, the Himalayan Region, and the northern part of South Asia (Blakelock 1951; Ma 2001). China hosts about 98 species that are widely distributed across the country, with more than 54 species endemic to China (Xiong et al. 2024; The Biodiversity Committee of the Chinese Academy of Sciences 2025; Hu et al. 2025; Ma et al. 2025; Qin et al. 2025). At present, Guizhou Province harbors approximately 58 species of the genus *Euonymus*, exhibiting remarkable diversity (Zhang et al. 2022).

The genus *Euonymus* is chiefly characterized by opposite leaves, axillary or terminal inflorescences, 4- or 5-merous flowers, stamens inserted on a fleshy annular disk, loculicidally dehiscent capsules, and arillate seeds. However, recent reports of cauliflory in *E. fangdingianus* (Xiong et al. 2024) have expanded the morphological understanding of the genus. A comprehensive taxonomic system for the genus *Euonymus*, comprising two subgenera, seven sections, and 14 series, was established by Blakelock (1951) based on characters such as winter bud morphology, aril morphology, and degree of fruit dehiscence. This system has been widely adopted by subsequent researchers (Cheng et al. 1999; Savinov and Baikov 2007). Morphologically, the genus *Euonymus* shares many traits with the genus *Glyptopetalum* Thwaites (Thwaites 1856). Phylogenetic analyses also suggest that these two genera form a monophyletic group. Nevertheless, because of limited taxon sampling and the short sequence lengths used in constructing these phylogenies, the results require further validation (Simmons et al. 2012; Li et al. 2014; Hu et al. 2022, 2025).

Renhuai City is located in northwest Guizhou Province, in the middle reaches of the Chishui River and on the northern flank of the West Dalou Mountain (Cheng et al. 2024). Notably, it is the first area in the province to have completed a comprehensive, jurisdiction-wide biodiversity survey. During a botanical survey conducted in October 2024 in Maoba Town and Maotai Town of Renhuai City, we collected this species at the fruiting stage. Its opposite leaves and capsules led to a preliminary identification as a member of Celastraceae. We revisited the site in June of the following year to observe the flowering phase and documented key diagnostic characters. Based on integrated evidence from field observations and detailed examination of voucher specimens, we propose this taxon as a new species of the genus *Euonymus*.

## Materials and methods

### Morphological characteristics

Specimens of this species were collected for morphological description and deposited as voucher specimens. Morphological characteristics were observed and measured from living plants. Comparisons with morphologically similar species were based on their type specimens and on morphological descriptions, photographs, and other specimens obtained through the following sources: Flora of China (http://www.efloras.org/), PPBC (https://ppbc.iplant.cn/), CVH (https://www.cvh.ac.cn/), JSTOR Global Plants (https://plants.jstor.org/), and iNaturalist (https://www.inaturalist.org/).

### Taxon sampling and phylogenetic inference

The phylogenetic analysis included 64 accessions, representing 59 taxa, including two species of *Celastrus* Linnaeus (Linnaeus 1753b) and one species of *Tripterygium* Hook.f. (Bentham and Hooker 1862) as outgroups. Seven species among the 56 entities included in the ingroup were documented as belonging to the genus *Glyptopetalum*. Information on taxa and GenBank accessions was summarized in Suppl. material 1. The internal transcribed spacer (ITS) sequences were selected for phylogenetic tree analysis, and both maximum likelihood (ML) and Bayesian inference (BI) methods were employed for tree construction. All analyses were performed using PhyloSuite (Zhang et al. 2020). For the BI analysis, the nucleotide substitution model for the data matrix was estimated using ModelFinder (Kalyaanamoorthy et al. 2017) under the corrected Akaike Information Criterion (AICc), and the GTR + I + G model was selected as the best-fit model. The ML analysis was conducted using IQ-TREE v1.4.2 (Nguyen et al. 2014), with the nucleotide substitution model selected via AIC in ModelFinder, resulting in the GTR + G model for the ITS region. The resulting phylogenetic trees were visualized using iTOL (https://itol.embl.de/).

### Taxonomic treatment

#### Euonymus maotaiensis

Taxon classificationPlantaeCelastralesCelastraceae

M.T.An & Xu Wu sp. nov.

17559297-DB24-5033-AD24-BECB84CAA617

urn:lsid:ipni.org:names:77376161-1

Figs 2, 3, 4

##### Type.

China. • Guizhou Province: Renhuai City, Maotai, 27°43'N, 106°13'E, alt. 500 m, 26 October 2024, *Ming-tai An, Feng Liu, Xu Wu, Jiang-hong Yu*, GZAC-WX-2594 (holotype: GZAC!).

**Figure 1.**
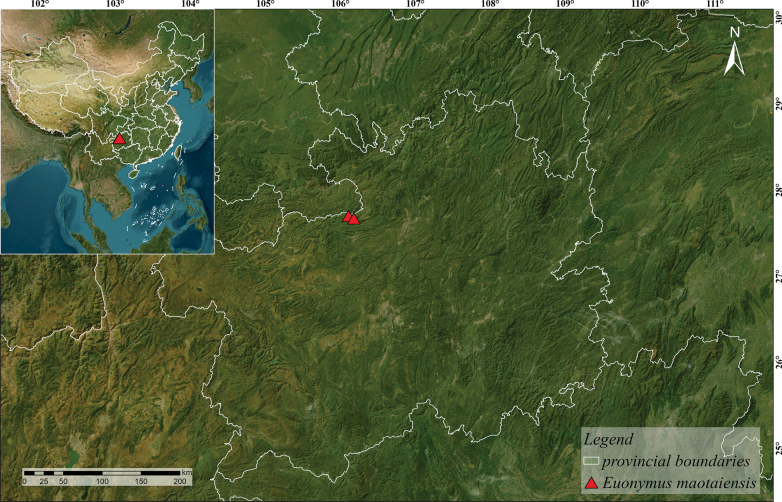
Geographical distribution of *Euonymus maotaiensis*.

**Figure 2.**
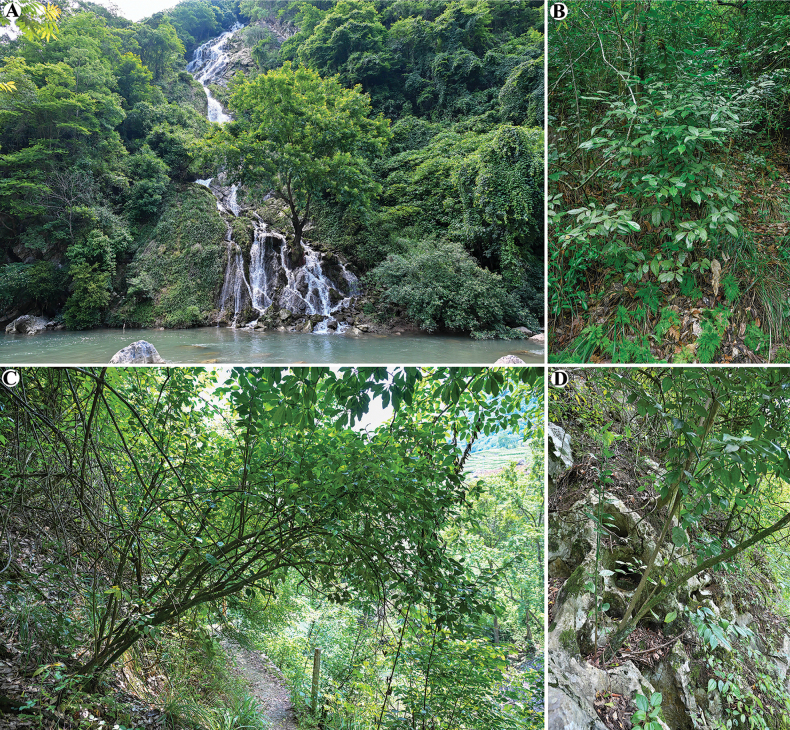
*Euonymus maotaiensis*. **A**. Habitat; **B, C**. Plant; **D**. Karst habitat.

**Figure 3.**
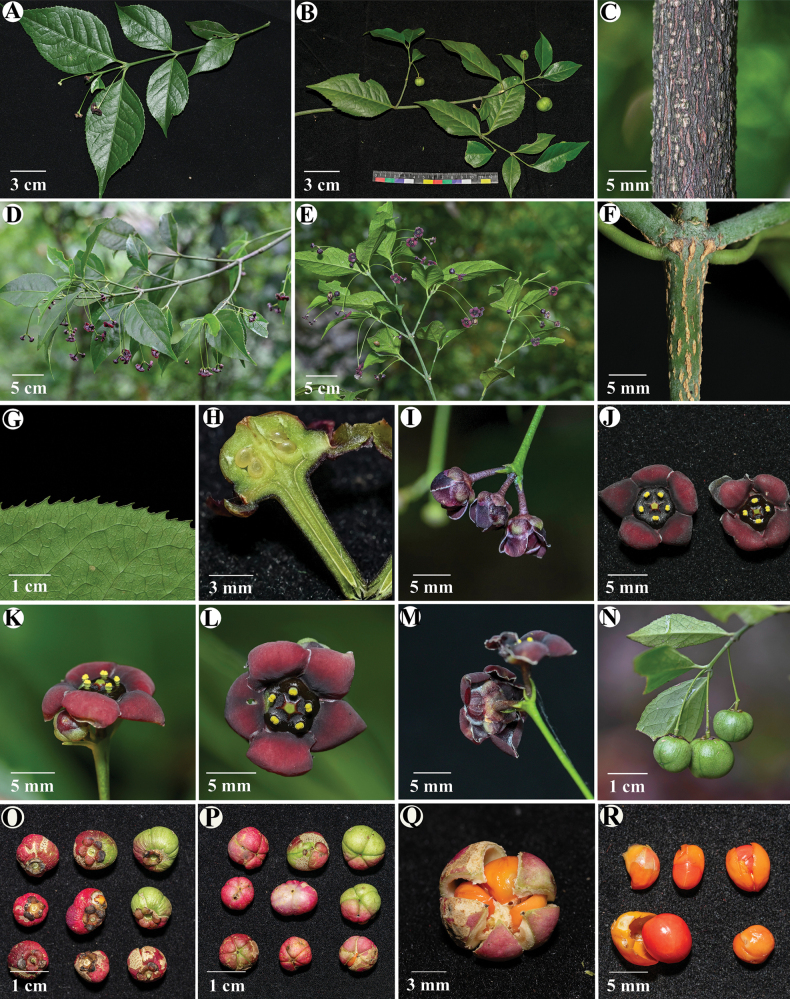
*Euonymus maotaiensis*. **A, D, E**. Branches with flowers; **B**. Branches with fruits; **C**. Lenticels on the old branches; **F**. Lenticels on the young branches; **G**. Serrate of leaf margin; **H**. Ovules; **I**. Buds; **J**. Number of perianth segments; **K**. Side view of flowers; **L**. Top view of flowers; **M**. Bottom view of flowers; **N**. Immature fruits; **O**. Top view of mature fruits; **P**. Bottom view of mature fruits; **Q**. Anatomical diagram of mature fruit; **R**. Seeds.

**Figure 4.**
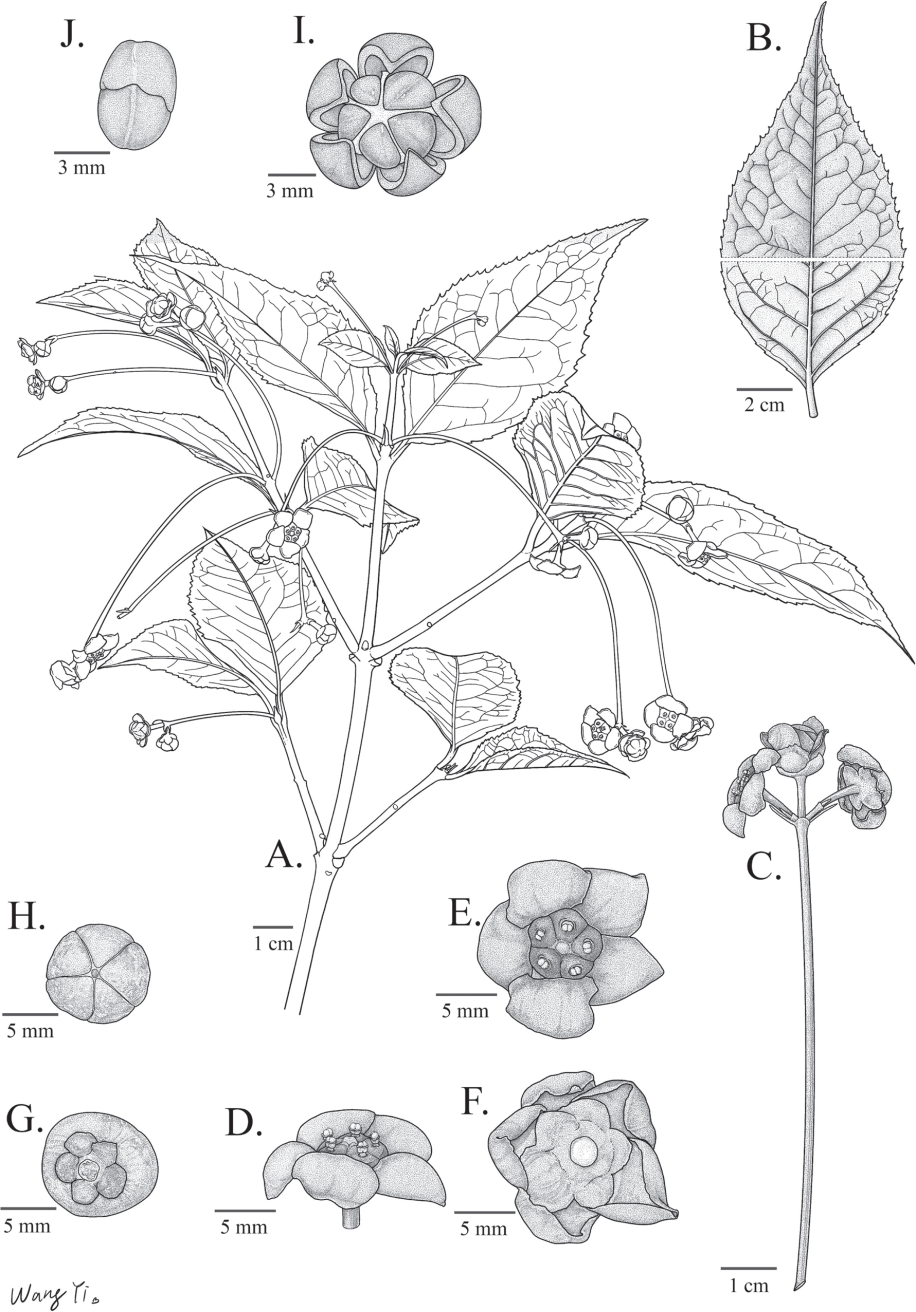
*Euonymus maotaiensis*. **A**. Branches with flowers; **B**. Leaf (adaxial surface above, abaxial surface below); **C**. Inflorescence; **D**. Side view of flowers; **E**. Top view of flowers; **F**. Bottom view of flowers; **G**. Top view of fruits; **H**. Bottom view of fruits; **I**. Anatomical diagram of fruit; **J**. Seeds. [Drawn by Yi Wang].

##### Diagnosis.

*E. maotaiensis* is morphologically similar to *E. chloranthoides* but can be distinguished by several key characteristics (Table 1): its broadly lanceolate leaves (as opposed to elliptic to oblong – elliptic), longer petioles (5–12 mm vs. 1–2 mm), longer peduncles (4–9 cm vs. 1–2 cm), 4- or 5-merous flowers (vs. strictly 4-merous), suborbicular petals with the upper half completely recurved abaxially (vs. planar petals), and indehiscent, 4–5 shallowly sulcate globose capsules (vs. 5-lobed capsules dehiscent to near the midpoint).

**Table 1.** Morphological comparison between *E. maotaiensis* and related species, including those distributed in the same area.

Character	*E. maotaiensis*	*E. chloranthoides*	*E. chengduensis*	*E. chinensis*
Branchlets	Slightly 4-angled	With four narrow ridges	Initially 4-angled, becoming terete at maturity	Slightly 4-angled
Leaf texture	Rigidly leathery	Thinly leathery	Thinly leathery	Leathery
Leaf shape	Broadly lanceolate (rarely lanceolate or elliptic)	Obovate, narrowly oblong-obovate, or elliptic to narrowly elliptic	Narrowly ovate, ovate-elliptic, or ovate-lanceolate	Obovate, elliptic, or oblong-broadly lanceolate
Leaf margin	Irregularly sharp serrate	Regularly coarsely spinose-dentate	More or less regularly spinose-dentate	Subentire
Petiole length	5–12 mm	1–2 mm	2–3 mm	6–10 mm
Inflorescence	2–3-flowered (rarely > 10)	3-flowered	3–7-flowered	3–15-flowered
Peduncle length	4–9 cm	1–2 cm	1.5–4.5 cm	2–4 cm
Flower merosity	Usually 5-merous (rarely four)	5-merous	4-merous	4-merous
Flower color	Purple-black	Purple-black	Deep red or dark red	White or yellowish-green
Petals	Broadly ovate, margin recurved, white-membranous, and irregularly undulate	Obovate, margin recurved with shallow irregular teeth	Ovate to broadly ovate, margin revolute, entire	Orbicular or obovate, margin flat, entire
Capsule	Globose, indehiscent, shallowly sulcate	5-lobed, dehiscent to near midpoint	Subglobose, indehiscent, shallowly sulcate	Triangular-ovoid, dehiscent, deeply 4-lobed, or broadly 4-angled

##### Description.

***Evergreen shrubs***, 1.5–4 m tall, glabrous throughout. ***Trunk*** and ***branches*** with conspicuous longitudinal lenticels; ***branchlets*** slightly 4-angled, green; ***Leaves*** opposite, rigidly coriaceous, extremely brittle when dry, usually broadly lanceolate, rarely lanceolate or elliptic, 6–12 × 2–6 cm, apex acuminate, base cuneate to broadly cuneate, margin irregularly sharp serrate; venation conspicuous on both surfaces, midvein raised abaxially, flush to slightly raised adaxially, lateral veins 6–9 pairs, impressed adaxially, prominent abaxially, anastomosing near the margin, reticulate with tertiary veinlets; ***petiole*** 0.5–1.2 cm long. ***Inflorescences*** mostly cymes with 2–3 flowers, rarely compound thyrses to > 10 flowers, extra-axillary or occasionally axillary; Peduncle elongate, 4–9 cm long; ***Flowers*** usually 5-merous, rarely 4-merous, purple-black, ca. 1 cm in diam; Pedicel usually purple-black (occasionally green), 0.5–0.9 cm long; ***Bracts*** usually longer than bracteoles, both linear, 0.4–1.1 cm long; ***Sepals*** suborbicular, purple-black or green, ca. half as long as petals, margin membranous; ***Petals*** suborbicular, purple-black, the upper half of the petal is completely reflexed and obscured from view, margin white-membranous and undulate, irregularly undulate; ***Staminal*** filaments Ca. 1 mm long; ***Pistil*** styles absent, stigma shortly columnar, disk flat, 4- 5-lobed. ovules erect to pendulous, 2 per locule. ***Capsule*** globose, red, 1–2 cm in diam, indehiscent with only 4–5 shallowly sulcate, usually 1 developed per inflorescence; fruiting pedicel slender, green; ***Seeds*** 4–5, bright orange-red in vivo.

##### Distribution and habitat.

This species is distributed in Maotai Town and Maoba Town, Renhuai City, Zunyi City, Guizhou Province (Fig. 1), at an elevation of ca. 500 m. It may be distributed in Sichuan Province and Chongqing City from the PPBC picture. It grows in well-lit broad-leaved forests along river valleys.

##### Phenology.

Flowering May – June; fruiting October – December.

##### Etymology.

“maotaiensis” refers to the discovery site of this species.

##### Local name.

Simplified Chinese: 茅台卫矛.

##### Conservation status.

We investigated potential habitats in the surrounding area and identified two populations totaling approximately 42 individuals. *Euonymus maotaiensis* primarily grows along forest edges, particularly on roadside margins, but was not observed in denser understory areas. In addition, a review of specimens and image databases from regions with similar habitats and elevations revealed that photographs taken by Li Xiaodong in Wuhan City, Hubei Province (PPBC id: 22545059–22545070) and those taken by Zhang Hua’an in Xuyong County, Luzhou City, Sichuan Province (PPBC id: 12794935, 12794949) represent *E. maotaiensis*. However, because a comprehensive investigation of the population status of *E. maotaiensis* has not yet been conducted, we recommend provisionally classifying it as Data Deficient (DD) according to IUCN criteria (IUCN 2022).

### Phylogenetic position analysis

**Figure 5.**
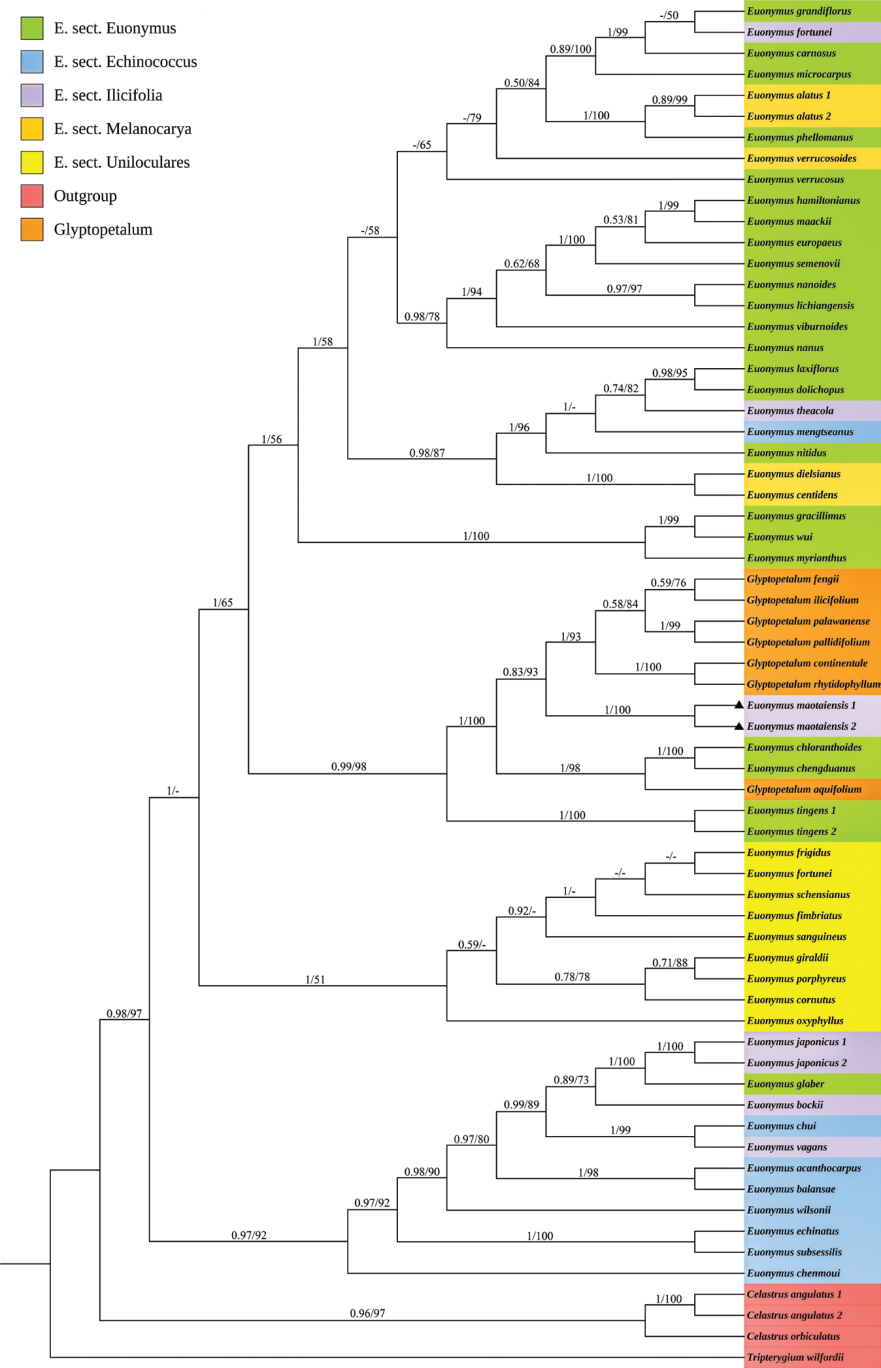
Bayesian inference and maximum likelihood phylogenetic tree based on nuclear internal transcribed spacer (ITS) sequences of 64 accessions representing 59 taxa of *Euonymus* and *Glyptopetalum*. Values above branches indicate Bayesian posterior probabilities (>0.50) / maximum likelihood bootstrap percentages (>50). Terminal nodes are shown in seven colors corresponding to the five sections of the genus *Euonymus* and *Glyptopetalum* described in “Flora of China” (Ma and Funston 2008), as well as the outgroup. Black triangle indicates *E. maotaiensis*.

Based on a dataset of ITS sequences, both Bayesian inference (BI) and maximum likelihood (ML) analyses indicate that sequences of the new species form a strongly supported monophyletic clade (BI = 1, ML = 100%). This new species is sister to a supported subclade (BI = 0.83, ML = 93%) containing six species of the genus *Glyptopetalum* (BI = 1, ML = 93%) (Fig. 5).

## Discussion

Morphologically, *E. maotaiensis* is clearly distinguishable from its close relatives, a conclusion further supported by phylogenetic analyses. The occasional misidentification of *E. maotaiensis* as *E. chloranthoides* in the PPBC database underscores their morphological similarity. However, key diagnostic characters evident in PPBC images – such as leaf shape, petiole length, degree of petal recurvature, and degree of capsule dehiscence – allow reliable differentiation between the two species. Nevertheless, it is often difficult to observe all of these distinguishing features simultaneously under field conditions, which can result in errors in identification.

Hu et al. (2022) proposed transferring *G. aquifolium* (Loes. & Rehder) C.Y. Cheng & Q.S. Ma to the genus *Euonymus*, reasoning that the genus *Glyptopetalum* is morphologically close to the genus *Euonymus*. The original distinction of the genus *Glyptopetalum* was based on its exclusively 4-merous flowers, a single pendulous ovule per locule of the ovary, and seeds with a branched raphe (Thwaites 1856; Ding Hou 1963; Simmons 2004; Ma and Funston 2008). However, other studies have suggested that *Glyptopetalum* should be included within a broadly defined genus, *Euonymus* (Li et al. 2014). Therefore, based on the diagnostic characteristics of *E. maotaiensis* – including 4- or 5-merous flowers, a flattened disk with non-rolled margins that do not enclose the ovary, and the presence of two ovules per locule – we classify it within the genus *Euonymus* in this study. Furthermore, based on features such as smooth branchlets without tubercles, 4- or 5-merous flowers, 2-locular anthers, and indehiscent capsules bearing 4–5 shallowly sulcate ribs, we assign it to sect. *Euonymus*.

## Supplementary Material

XML Treatment for Euonymus maotaiensis

## Additional information

### Use of AI

No use of AI was reported.

### Author ORCIDs

Xu Wu https://orcid.org/0009-0004-7935-6057

Feng Liu https://orcid.org/0009-0003-7031-4494

Ming-Tai An https://orcid.org/0000-0003-3886-0287

Jiang-Hong Yu https://orcid.org/0000-0003-1765-8557

Wan-Ying Yang https://orcid.org/0009-0004-8651-5634

### Data availability

All of the data that support the findings of this study are available in the main text or Supplementary Information.

## Supplementary materials

Supplementary material 1 Voucher information for taxa used·in this·study Data type pdf https://binary.pensoft.net/file/1525300This dataset is made available under the Open Database License (http://opendatacommons.org/licenses/odbl/1.0/). The Open Database License (ODbL) is a license agreement intended to allow users to freely share, modify, and use this Dataset while maintaining this same freedom for others, provided that the original source and author(s) are credited. Xu Wu, Feng Liu, Ming-Tai An Jiang-Hong Yu, Wan-Ying Yang
