# Supplementary material for: Euonymus maotaiensis M.T.An & Xu Wu, sp. nov., a new species of Euonymus (Celastraceae) from southwest China
Source: PhytoKeys. 2026 Feb 2;270:143–53. doi: 10.3897/phytokeys.270.171340 (PMC12887579; doi:10.3897/phytokeys.270.171340)
Supplement: Supplementary material 1 — Voucher information for taxa used·in this·study [file phytokeys-270-143_article-171340__-s001.pdf]

S 1. Voucher information for taxa used·in this·study

rank	Group type	Genus	species	GenBank
1	outgroup	Celastrus	1. *Celastrus angulatus* Maxim.	EU328759.1
2	outgroup	Celastrus	2. *Celastrus angulatus* Maxim.	MH711208.1
3	outgroup	Celastrus	*Celastrus orbiculatus* Thunb.	MN647936.1
4	outgroup	Tripterygium	*Tripterygium wilfordii* Hook. f.	HM115962.1
5	ingroup	Euonymus	*Euonymus acanthocarpus* Franch.	KF282154.1
6	ingroup	Euonymus	*Euonymus alatus* (Thunb.) Siebold	KF282156.1
7	ingroup	Euonymus	*Euonymus alatus* (Thunb.) Siebold	MH711819.1
8	ingroup	Euonymus	*Euonymus aquifolium* (Loes. & Rehder) C. Y. Cheng & Q. S. Ma	OK172405.1
9	ingroup	Euonymus	*Euonymus balansae* Sprague	KF282157.1
10	ingroup	Euonymus	*Euonymus bockii* Loes. ex Diels	KF282158.1
11	ingroup	Euonymus	*Euonymus carnosus* Hemsl.	KF282160.1
12	ingroup	Euonymus	*Euonymus centidens* H. Lév.	KF282161.1
13	ingroup	Euonymus	*Euonymus chengduanus* J. Hu & H. He	OR725028.1
14	ingroup	Euonymus	*Euonymus chenmoui* W. C. Cheng	KF282162.1
15	ingroup	Euonymus	*Euonymus chloranthoides* Yang	OR725029.1
16	ingroup	Euonymus	*Euonymus chui* Handel-Mazzetti	KF282163.1
17	ingroup	Euonymus	*Euonymus cornutus* Hemsl.	MH710947.1
18	ingroup	Euonymus	*Euonymus dielsianus* Loes. & Diels	KF282167.1
19	ingroup	Euonymus	*Euonymus dolichopus* Merr. ex J. S. Ma	KF282168.1
20	ingroup	Euonymus	*Euonymus echinatus* Wall. ex Roxb.	KF282169.1
21	ingroup	Euonymus	*Euonymus europaeus* L.	HQ393713.1
22	ingroup	Euonymus	*Euonymus fimbriatus* Wall.	HQ393710.1
23	ingroup	Euonymus	*Euonymus fortunei* (Turcz.) Hand.-Mazz.	KF282177.1
24	ingroup	Euonymus	*Euonymus frigidus* Wall.	MW382667.1
25	ingroup	Euonymus	*Euonymus giraldii* Loes. ex Diels	MW382662.1
26	ingroup	Euonymus	*Euonymus glaber* Roxb.	HQ393703.1
27	ingroup	Euonymus	*Euonymus gracillimus* Hemsl.	KF282173.1
28	ingroup	Euonymus	*Euonymus grandiflorus* Wall.	HQ393711.1
29	ingroup	Euonymus	*Euonymus hamiltonianus* Wall.	HQ393724.1
30	ingroup	Euonymus	*Euonymus japonicus* 1 Thunb.	HQ393700.1
31	ingroup	Euonymus	*Euonymus japonicus* 2 Thunb.	KP214503.1
32	ingroup	Euonymus	*Euonymus laxiflorus* Blume ex Miq.	KF282179.1
33	ingroup	Euonymus	*Euonymus lichiangensis* W. W. Sm.	MH117525.1
34	ingroup	Euonymus	*Euonymus maackii* Rupr.	MT923464.1
35	ingroup	Euonymus	1. *Euonymus maotaiensis* L.	-
36	ingroup	Euonymus	2. *Euonymus maotaiensis* L.	-

37	ingroup	Euonymus	*Euonymus mengtseanus* (Loes.) Sprague	KF282182.1
38	ingroup	Euonymus	*Euonymus microcarpus* (Oliv. ex Loes.) Sprague	KF282183.1
39	ingroup	Euonymus	*Euonymus myrianthus* hemsl.	HQ393721.1
40	ingroup	Euonymus	*Euonymus nanoides* Loes. & Rehder	KF282187.1
41	ingroup	Euonymus	*Euonymus nanus* M. Bieb.	HQ393709.1
42	ingroup	Euonymus	*Euonymus nitidus* Benth.	KP092574.1
43	ingroup	Euonymus	*Euonymus oxyphyllus* Miq.	KF282190.1
44	ingroup	Euonymus	*Euonymus phellomanus* Loes. ex Diels	HQ393718.1
45	ingroup	Euonymus	*Euonymus porphyreus* Wall.	KF282196.1
46	ingroup	Euonymus	*Euonymus sanguineus* Loes. ex Diels	MH808347.1
47	ingroup	Euonymus	*Euonymus schensianus* Maxim.	MH710761.1
48	ingroup	Euonymus	*Euonymus semenovii* Regel & Herder	KF282197.1
49	ingroup	Euonymus	*Euonymus subsessilis* Wall. ex Roxb.	KF282203.1
50	ingroup	Euonymus	*Euonymus theacola* C. Y. Cheng ex T. L. Xu & Q. H. Chen	KF282204.1
51	ingroup	Euonymus	1. *Euonymus tingens* Wall.	HQ393716.1
52	ingroup	Euonymus	2. *Euonymus tingens* Wall.	MH117530.1
53	ingroup	Euonymus	*Euonymus vagans* Wall.	HQ393720.1
54	ingroup	Euonymus	*Euonymus verrucosoides* Loes.	KF282207.1
55	ingroup	Euonymus	*Euonymus verrucosus* Scop.	HQ393717.1
56	ingroup	Euonymus	*Euonymus viburnoides* Prain	KF282208.1
57	ingroup	Euonymus	*Euonymus wilsonii* Sprague	KF282209.1
58	ingroup	Euonymus	*Euonymus wui* J. S. Ma	JQ424145.1
59	ingroup	Glyptopetalum	*Glyptopetalum continentale* (Chun & F. C. How) C. Y. Cheng & Q. S. Ma	KF282210.1
60	ingroup	Glyptopetalum	*Glyptopetalum fengii* (Chun & F. C. How) Ding Hou	MZ568391.1
61	ingroup	Glyptopetalum	*Glyptopetalum ilicifolium* (Franch.) C. Y. Cheng & Q. S. Ma	OM985812.1
62	ingroup	Glyptopetalum	*Glyptopetalum palawanense* Thwaites	HQ393705.1
63	ingroup	Glyptopetalum	*Glyptopetalum pallidifolium* (Hayata) Q.R.Liu & S.Y.Meng	KF282192.1
64	ingroup	Glyptopetalum	*Glyptopetalum rhytidophyllum* (Chun & F. C. How) C. Y. Cheng	HQ393706.1
